# CXCR4 knockdown prevents inflammatory cytokine expression in macrophages by suppressing activation of MAPK and NF-κB signaling pathways

**DOI:** 10.1186/s13578-019-0315-x

**Published:** 2019-07-03

**Authors:** Xue Tian, Guogang Xie, Hui Xiao, Fengming Ding, Wuping Bao, Min Zhang

**Affiliations:** Department of Respiratory and Critical Care Medicine, Shanghai General Hospital, Shanghai Jiao Tong University School of Medicine, 100 Haining Road, Shanghai, 200080 People’s Republic of China

**Keywords:** CXCR4, IL-6, TNF-α, Macrophage, MAPK, NF-κB

## Abstract

**Background:**

Recent evidence has shown that C-X-C chemokine receptor type 4 (CXCR4) plays a crucial role in acute lung injury (ALI). Macrophages are key factors in the pathogenesis of ALI. The aim of this study was to investigate the role of CXCR4 in macrophages after lipopolysaccharide (LPS) stimulation and confirm that CXCR4 knockdown can inhibit inflammatory cytokines by suppressing mitogen-activated protein kinase (MAPK) and nuclear factor-κB (NF-κB) signaling pathway activation.

**Results:**

In this study, we found that CXCR4 expression in lung tissue of ALI was significantly increased using immunofluorescence. We also found that the expression of CXCR4 in macrophages sorted from bronchoalveolar lavage fluid (BALF) of ALI was obviously upregulated through RT-qPCR. After CXCR4 knockdown using siRNA, we found that the expression of interleukin-6 (IL-6) and tumor necrosis factor alpha (TNF-α) was obviously down regulated in macrophages. Additionally, the phosphorylation of p38, Erk, and p65 was significantly decreased after CXCR4 knockdown through western blotting.

**Conclusions:**

Taken together, the present study suggests that CXCR4 knockdown may inhibit inflammatory cytokine expression in macrophages by suppressing MAPK and NF-κB signaling pathway activation. Therefore, CXCR4 knockdown may have potential clinical value in treating ALI.

## Background

Acute lung injury (ALI) is a life-threatening disease characterized by an increased permeability of the alveolar-capillary barrier, resulting in lung edema with protein-rich fluid and consequently in impaired arterial oxygenation. Lipopolysaccharide (LPS) inhalation is equal to human Gram-negative ALI, leading to recruitment of neutrophils, pulmonary edema, and finally impairment of gas exchange [1, 2]. Intrapulmonary inflammatory response caused by a large number of inflammatory cells into the lung is an important feature of ALI [3]. ALI can also induce activation of more signal transduction pathways [4, 5] and cytokine secretion, such as tumor necrosis factor alpha (TNF-α), interleukin (IL)-1, interleukin (IL)-10, and interleukin (IL)-6 [6].

Macrophages play an important role in ALI, which can identify pathogen-associated molecular patterns and trigger congenital immunities to produce host defense [7, 8]. The activation of alveolar macrophages and the release of inflammatory cytokines are the important causes of inflammation and development [9]. Macrophages were polarized to M1 macrophages induced by LPS stimulation and secreted a large number of inflammatory mediators and cytokines, such as tumor necrosis factor-alpha (TNF-α), interleukin-1beta (IL-1β), interleukin-6 (IL-6), inducible nitric oxide synthase (iNOS), and macrophage migration inhibitory factor (MIF). TNF-α can directly damage pulmonary vascular endothelium cells, lead to capillary endothelial permeability, and cause pulmonary edema. IL-6 can predict the severity of ALI [10].

Chemokine receptors are critical for directed migration of leukocytes from the circulation to sites of inflammation [11]. CXCL12 (C-X-C motif chemokine ligand 12), is a chemokine produced constitutively in the bone marrow, which must be bound to CXCR4 (C-X-C chemokine receptor type 4) expressed by leukocytes and hematopoietic stem/progenitor cells. CXCL12 must bind and interact with CXCR4 to initiate downstream signaling pathways [12]. CXCR4 is an alpha chemokine receptor. CXCR4 signaling has been observed to play a key role in several pathological processes, including invasion of pancreatic cancer, Ewing sarcoma, esophageal cancer, and inflammatory disease. The mechanism of CXCR4 signaling pathway-mediated inflammatory responses may affect the efficient chemotaxis function of inflammatory cells, such as neutrophils, lymphocytes, and monocytes. CXCR4 can activate endothelial cells and promote inflammatory cells passing through vascular endothelium to the inflammation site [13, 14]. Some studies revealed that in patients with ALI and in LPS-induced ALI mouse models, the expression of CXCR4 was significantly increased in lung tissue [15]. Additionally, CXCR4 was further confirmed to produce the chemotactic effect of neutrophils.

Therefore, this study is the first to demonstrate that the expression of CXCR4 was significantly increased in lung tissue of LPS-induced ALI mice. We further found that CXCR4 expression was also obviously increased in RAW264.7 macrophages with LPS stimulation. However, after CXCR4 knockdown using siRNA, the expression of IL-6 and TNF-α was decreased, but IL-10 expression was increased. In addition, we also demonstrated that the activation of mitogen-activated protein kinase (MAPK) and nuclear factor kappa beta (NF-κB) was suppressed after CXCR4 knockdown. The aim of this study was to determine whether CXCR4 can regulate inflammation of ALI through macrophages. Another aim of this study was to determine whether CXCR4 maybe the therapeutic target of ALI in the near future.

## Materials and methods

### Reagents

Antibody used for flow cytometry: CXCR4-PE was purchased from Biolegend (San Diego, CA, USA). Antibodies against GAPDH, Erk1/2, p-Erk1/2, JNK, p-JNK, p38, p-p38, p-65 and p-p65 were from Cell Signaling Technology (Danvers, MA, USA). LPS was from InvivoGen (San Diego, CA, USA). Dulbecco’s modified Eagle’s medium (DMEM) and bovine serum were from Gibco (Carlsbad, CA, USA). The PrimeScript RT-PCR Kit and SYBR Premix Ex Taq kit were from Takara Biology Company (Shiga, Japan). Lipofectamine™ 2000 Transfection Reagent was from Thermo Fisher (Pittsburgh, PA, USA).

### Cell culture

The macrophage cell line RAW264.7 was obtained from American Type Culture Collection (ATCC, USA) and cultured in endotoxin-free DMEM containing 10% fetal bovine serum (FBS, Gibco, USA).

### Mice

C57BL/6 mice were purchased from Shanghai Laboratory Animal Center (Shanghai, China). All mice were housed in pathogen-free condition with standard laboratory chow and water ad libitum in Laboratory Animal Center of Shanghai General Hospital, Shanghai Jiao Tong University School of Medicine. All animal experiments were carried out following the guidelines of the institutional Animal Ethics Committee of Shanghai General Hospital, Shanghai Jiao Tong University School of Medicine.

### RNA-mediated interference

The siRNA specific to murine CXCR4: GGTTACCAGAAGCTAA and silencer negative control siRNA (Ruibo Biology Company, China) were transfected into mouse peritoneal macrophages using Lipofectamine 2000 Transfection Reagent (ThermoFisher, USA) according to the manufacturer’s instructions.

### RT-PCR or quantitative real-time PCR analysis of gene expression

Total RNAs were extracted using Trizol Reagent (Takara, Japan) and the reverse transcription was performed using Prime-Script™ RT Reagent Kit according to the manufacturer’s instructions (Takara, Japan). The expression of mRNA encoding CXCR4 (forward: GAGGCCAAGGAAACTGCTG, reverse: GCGGTCACAGATGTACCTGTC), IL-6 (forward: TAGTCCTTCCTACCCCAATTTCC, reverse: TTGGTCCTTAGCCACTCCTTC), TNF-α (forward: CAGGCGGTGCCTATGTCTC; reverse: CGATCACCCCGAAGTTCAGTAG), and IL-10 (forward: GCTCTTACTGACTGGCATGAT, reverse: CGCAGCTCTAGGAGCATGTG) was determined by quantitative real-time PCR (Q-PCR) and was normalized to the expression of glyceraldehyde 3-phosphate dehydrogenase (GAPDH). Q-PCR was conducted in the Light Cycler Quantitative PCR Apparatus (Illumina, USA) using the SYBR Green Master Mix (Takara, Japan).

### FACS analysis of surface molecule expression

RAW264.7 cells were exposed to LPS (100 ng/ml) for 12 h and stained with a PE-labeled anti-CXCR4 (Biolegend, USA). Fluorescence-activated cell sorting (FACS) analysis was performed on a flow cytometer (Becton–Dickinson, USA) using CellQuest software (BD Biosciences, USA). Results are expressed as mean fluorescent density (MFI).

### Western blot analysis

RAW264.7 cells were washed twice with cold phosphate-buffered saline (PBS, pH = 7.0), and lysed in radioimmunoprecipitation (RIPA) buffer [Cell Signaling Technology (CST), USA] supplemented with protease inhibitors (Roche, USA). The protein concentration of each sample was assayed using the bicinchoninic acid method (BCA kit) (Pierce, Rockford, IL, USA). Equal amounts of protein (60 μg) were subjected to sodium dodecyl sulfate polyacrylamide gel electrophoresis (SDS-PAGE) on 12.5% gel. Then, the protein was blotted onto a NC membrane. After blocking with 5% non-fat milk in 20 mM of tris-buffered saline (TBS) with 0.1% Tween for 1 h at room temperature with shaking, they were incubated with the indicated primary antibodies at 4 °C overnight, followed by the appropriate fluorescent secondary antibodies (1:5000 dilution) for 2 h at room temperature. The immune-reactive proteins were detected using the Odyssey laser digital imaging system (Gene Company). Primary antibodies employed in this study included anti-GAPDH (1:1000, CST, USA), anti-p65 (1:1000, CST, USA), anti-Erk1/2 (1:1000, CST, USA), anti-JNK (1:1000, CST, USA), anti-p38 (1:1000, CST, USA), anti-p-p65 (1:1000, CST, USA), anti-p-Erk1/2 (1:1000, CST, USA), anti-p-JNK (1:1000, CST, USA), anti-p-p38 (1:1000, CST, USA) and anti-CXCR4 (1:1000, Thermo Fisher, USA).

### LPS-induced ALI model

Eight-week-old male wild-type C57BL/6 mice were anesthetized by inhaling isoflurane (100 mg/kg), followed by intranasal (i.n.) administration of 50 μl of LPS (100 mg/kg in PBS). Control mice received i.n. instillation of 50 μl of PBS. The mice were killed and perfused using aortic phlebotomy at 48 h after LPS administration.

### Immunofluorescence

Four-micrometer sections of mouse lung were fixed with 4% paraformaldehyde (PFA), embedded in optimal cutting temperature medium, and incubated in blocking buffer (1 h, 5% wt/vol bovine serum albumin (BSA), 1% skim milk, 0.05% Triton X-100 in PBS). Sections were then incubated overnight with rabbit anti-CXCR4 antibody (1:1000, CST, USA) and then washed in PBS (0.05% Tween 20). Sections were washed and then incubated with Cy3-conjugated secondary antibodies (1:300, CST, USA) for 1 h and then washed. Nuclei were stained with 4′,6-diamidino-2-phenylindole (DAPI, 1:1000, Sigma, USA) for 10 min. After the sections were coverslipped with 50% glycerinum, the images were captured using fluorescence microscope (DP7, Olympus, Japan).

### Statistical analysis

Data are presented as mean ± SEM. All data were tested for significance using GraphPad Prism software (GraphPad Software) with statistical significance at P < 0.05, with one-way analysis of variance (ANOVA) using the Tukey post hoc test for comparing multiple data sets and using unpaired T-test for comparing two sets of data.

## Results

### CXCR4 expression was significantly increased in LPS-induced ALI

**Fig. 1:**
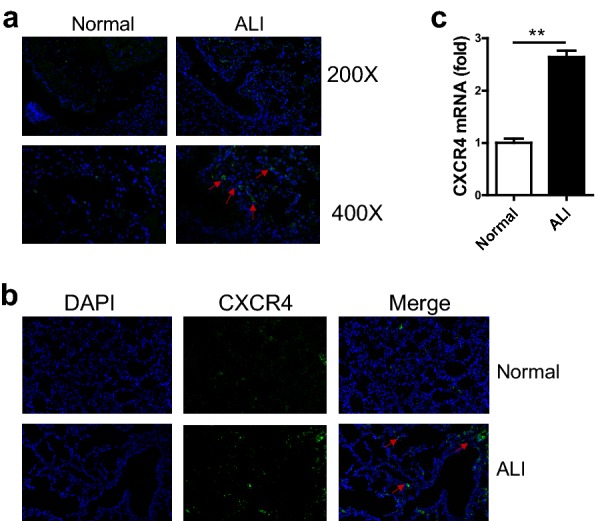
The expression of CXCR4 was detected in ALI lung tissue and alveolar macrophages. **a** Immunofluorescence analyzed a number of macrophages in ALI lung tissue. **b** Immunofluorescence analyzed the expression of CXCR4 in ALI lung tissue. **c** RT-qPCR analyzed CXCR4 expression in alveolar macrophages. CXCR4 in macrophage of lung tissue in control group (×200) and ALI group (×200). Data shown represent at least three independent experiments. *p < 0.05, **p < 0.01, and ***p < 0.001 compared with control group. Error bars represent SD

To investigate the function of CXCR4 in ALI, we first established an ALI mouse model using LPS induced for 48 h. We found that large numbers of macrophages infiltrated the lung tissue of ALI mice using immunofluorescence (Fig. 1a). This result suggested that macrophages maybe play a key role in ALI. Additionally, we found that the expression of CXCR4 in ALI lung tissue was also increased through immunofluorescence compared with that of the normal group (Fig. 1b). To examine whether the expression of CXCR4 in macrophages was also increased, we sorted macrophages from bronchoalveolar lavage fluid (BALF) of ALI and normal mice, and mRNA was extracted immediately. RT-qPCR was used to analyze the expression of CXCR4. The result showed that CXCR4 expression was also significantly increased in macrophages from BALF of ALI mice (Fig. 1c). Altogether, these data demonstrated that CXCR4 expression was significantly increased in LPS-induced ALI and alveolar macrophages.

### CXCR4 expression was significantly increased in macrophage cell line RAW264.7 after LPS stimulation

**Fig. 2:**
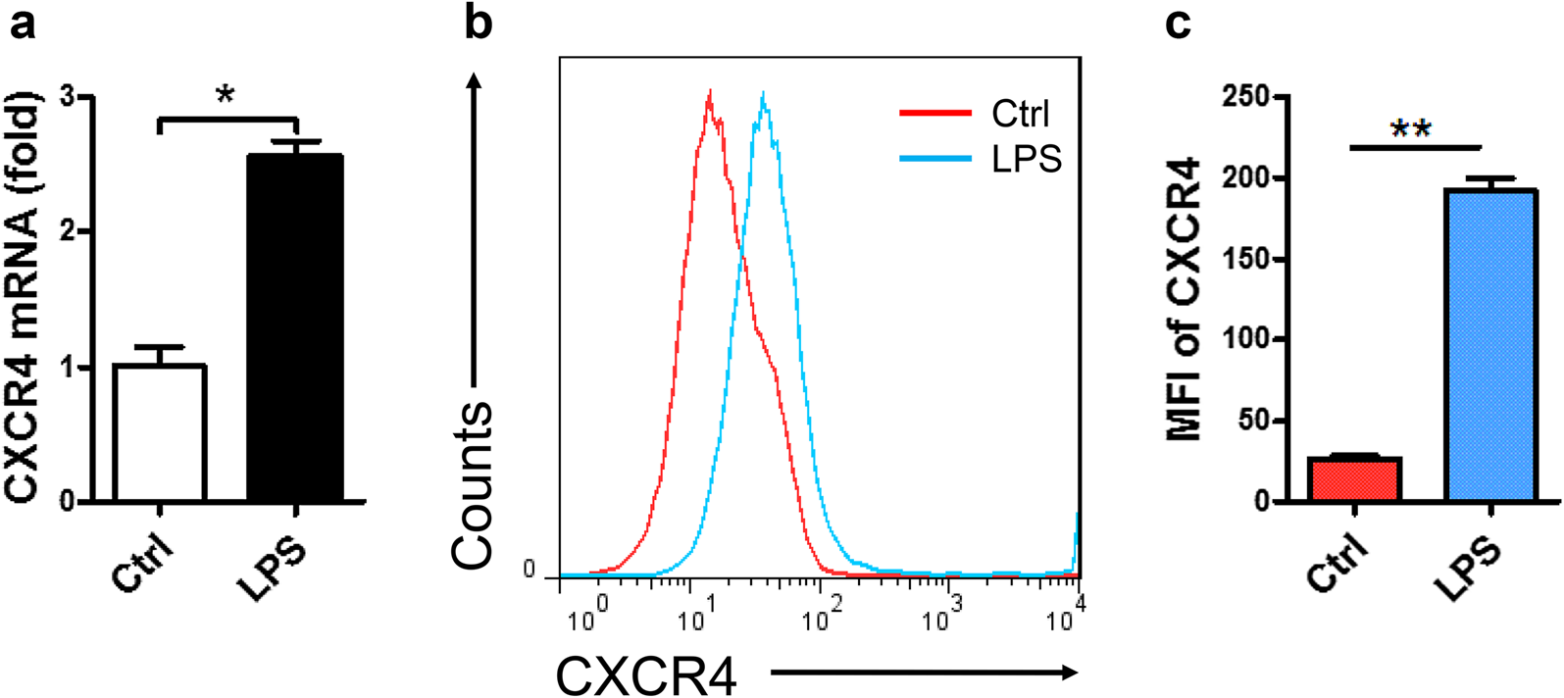
The expression of CXCR4 was detected in macrophage cell line RAW264.7. **a** RT-qPCR analyzed the expression of CXCR4 in macrophage cell line RAW264.7. **b**, **c** FACS analyzed the expression of CXCR4 in macrophage cell line RAW264.7. **a**–**c** CXCR4 expression in LPS stimulation group and control group. Data shown represent at least three independent experiments. *p < 0.05, **p < 0.01, and ***p < 0.001 compared with the control group. Error bars represent SD

To verify the above data, macrophage cell line RAW264.7 was used and stimulated with LPS for 6 or 12 h. We found that the expression of CXCR4 was obviously increased using RT-qPCR (Fig. 2a). We further used FACS to examine these results and found that the expression of CXCR4 in RAW264.7 was also quickly and significantly increased after LPS stimulation (Fig. 2b, c). Altogether, these data indicated that CXCR4 expression was significantly increased in RAW264.7 after LPS stimulation.

### CXCR4 knockdown in macrophage cell line RAW264.7

**Fig. 3:**
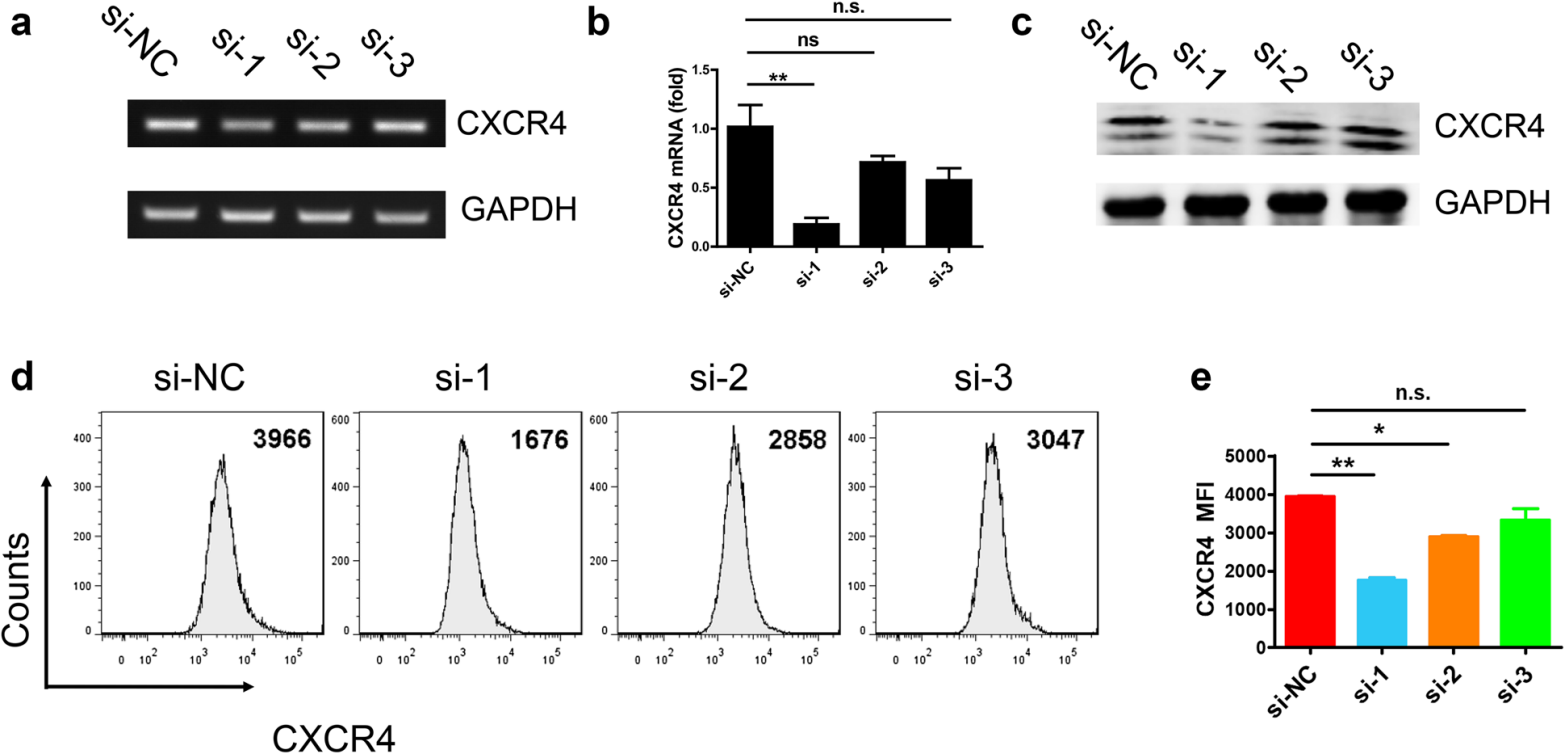
CXCR4 knockdown in RAW264.7 cells. **a** Interference effect of different siRNA sequence of CXCR4 (si-1, si-2, si-3) were observed using RT-PCR. **b** Interference effect in different CXCR4 siRNA by RT-qPCR. **c** Western blotting analyzed CXCR4 expression of different interference sequence. **d**, **e** FACS analyzed CXCR4 expression of different interference sequence. Data shown represent at least three independent experiments. *p < 0.05, **p < 0.01, and ***p < 0.001 compared with control group. Error bars represent SD

To knockdown CXCR4, we designed three different small interfering RNA (si-1, si-2, and si-3) of CXCR4. RT-qPCR, western blotting and FACS were used to analyze which siRNA had the best interference effect. We first found that the expression of CXCR4 in RAW264.7 was significantly decreased using si-1 through RT-qPCR (Fig. 3a, b). To further verify whether si-1 had the best interference, we used western blotting to detect CXCR4 expression in RAW264.7. The data showed the expression of CXCR4 was also obviously decreased (Fig. 3c). Meanwhile, we also used the FACS to exam this result, the data showed that the expression of CXCR4 was significant decreased with down regulated MFI of CXCR4 (Fig. 3d, e). Collectively, si-1 had the best interference effect compared to the others.

### CXCR4 knockdown inhibits the expression of IL-6 and TNF-α

**Fig. 4:**
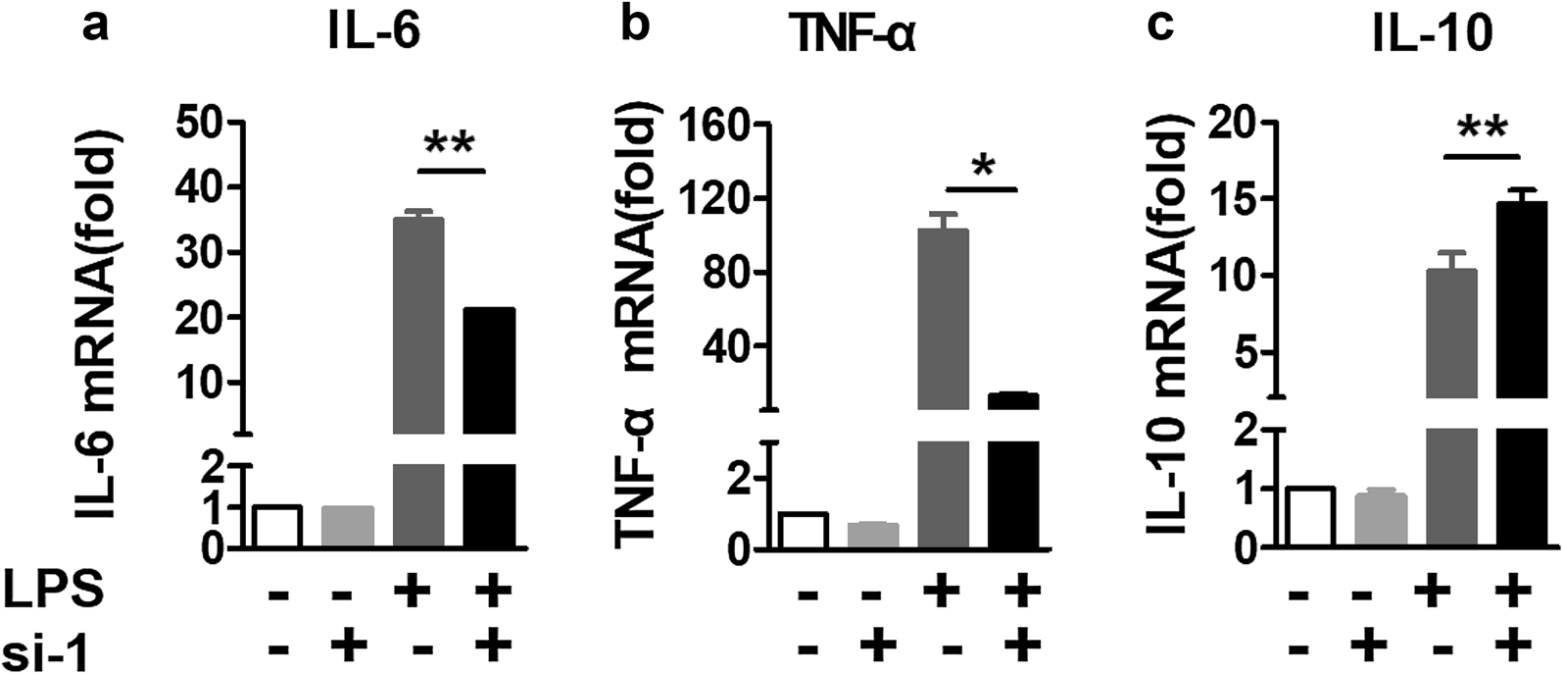
The expression of IL-6, TNF-α, and IL-10 was detected in CXCR4 knockdown RAW264.7 cells after LPS stimulation. **a**–**c** RAW264.7 cells were stimulated with or without LPS for 12 h and mRNA was extracted immediately. RT-qPCR analyzed the expression of IL-6, TNF-α, and IL-10. Data shown represent at least three independent experiments. *p < 0.05, **p < 0.01, and ***p < 0.001 compared with control group. Error bars represent SD

According to the above data, we chose si-1 as the best interfering sequence to knockdown CXCR4. To investigate the function of CXCR4 in macrophages, si-1 was transfected into RAW264.7 using Lipofectamine 2000 transfection reagent for 24 h. After that, we detected the expression of IL-6, TNF-α, and IL-10 using RT-qPCR. There was no obvious change in the expression of IL-6, TNF-α, and IL-10 with CXCR4 siRNA compared with no CXCR4 siRNA in RAW264.7 without LPS stimulation. However, the expression of IL-6 and TNF-α was significantly decreased compared no control group with CXCR4 konckdown group after LPS stimulation, but the expression of IL-10 was obviously increased in CXCR4 knockdown group (Fig. 4a–c). Altogether, these data suggested that CXCR4 knockdown suppresses the expression of IL-6 and TNF-α and promotes the expression of IL-10.

### CXCR4 knockdown prevents the activation of MAPK and NF-κB signaling pathways

**Fig. 5:**
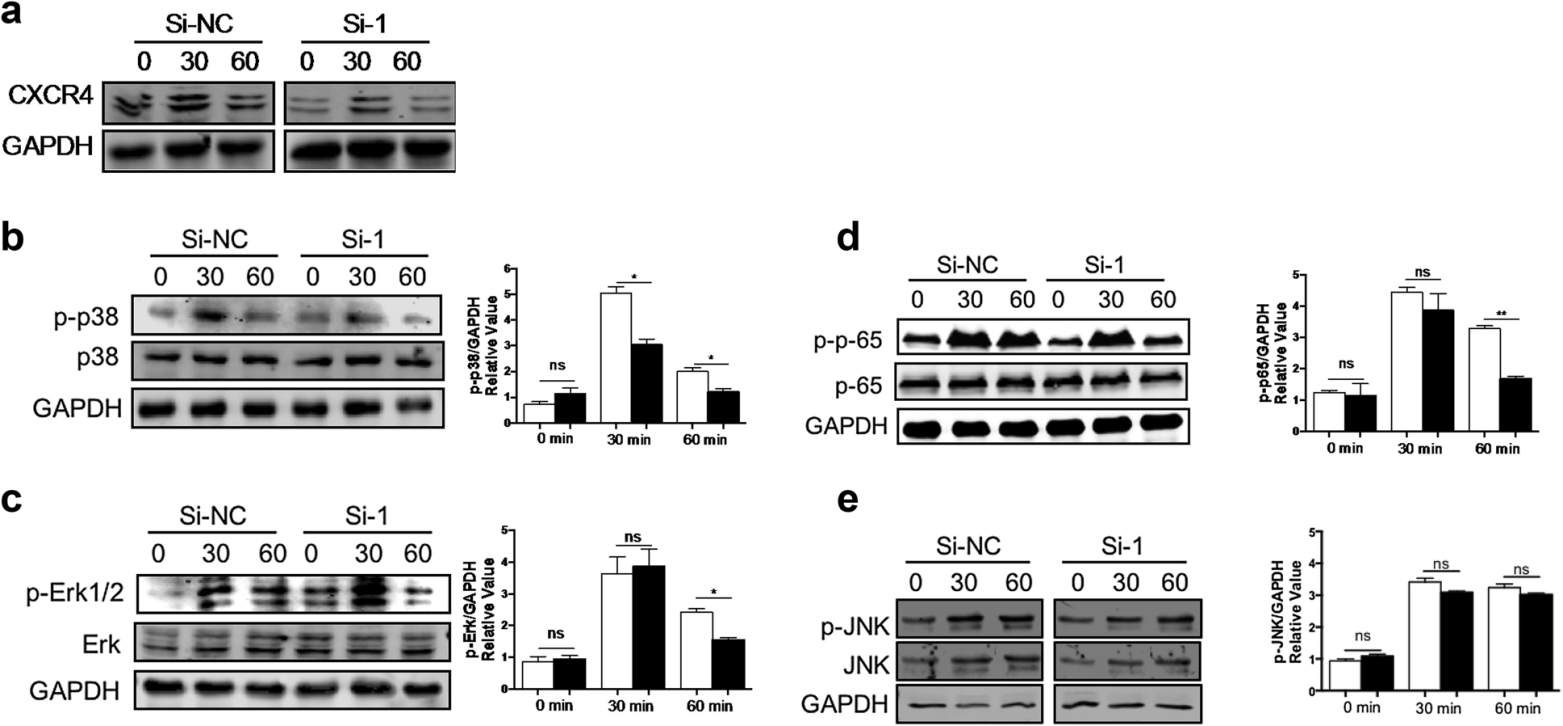
The activation of the MAPK and NF-κB signaling pathways was detected in CXCR4 knockdown RAW264.7 cells. **a** RAW264.7 cells were stimulated with or without LPS for 30 min or 60 min. Cells were harvested and lysed, followed by western blotting using CXCR4 antibody. **b**–**e** RAW264.7 cells were stimulated with or without LPS for 30 min or 60 min. Cells were harvested and lysed, followed by western blotting using p-p38, p-Erk1/2, p-JNK, and p-p65. Data shown represent at least three independent experiments. *p < 0.05, **p < 0.01, and ***p < 0.001 compared with control group. Error bars represent SD

To investigate the mechanism by which CXCR4 knockdown decreases the expression of IL-6 and TNF-α in macrophages, we first detected the downstream MAPK signaling pathways of LPS. The data showed that the CXCR4 was decreased in this experiment using the si-1 siRNA (Fig. 5a). Next we found that the levels of p-p38 and p-Erk were significantly decreased in the CXCR4 siRNA group after LPS stimulation for 60 min (Fig. 5b, c). To investigate whether CXCR4 affects NF-κB signaling pathway, we detected the phosphorylation of p65 in the downstream of NF-κB signaling pathway. The results showed that the phosphorylation level of p65 after LPS stimulation for 60 min in CXCR4 siRNA group was also obviously decreased compared to that of the control group (Fig. 5d). However, there was no obvious difference in the level of p-JNK between CXCR4 siRNA and control (Fig. 5e). Altogether, these results demonstrated that CXCR4 knockdown suppresses MAPK and NF-κB signaling pathway activation.

## Discussion

ALI develops due to uncontrolled systemic inflammatory responses to direct or indirect lung injury. At the cellular level, ALI manifests as infiltration, activation of inflammatory cells, and damage of vascular endothelial cells [16]. At the molecular level, it is characterized by activation of NF-κB and overexpression of numerous inflammatory factors and chemokines. The main inflammatory cells include neutrophils, alveolar macrophages, and vascular endothelial cells. These inflammatory cells can secrete different cytokines, such as IL-1β, TNF-α, IL-6, and IL-10 [17].

Macrophages not only have phagocytic functions, but also have secretory functions. Currently, there is some evidence suggesting that macrophages, including resident alveolar macrophages and recruited macrophages from the blood, are key factors in the pathogenesis of ALI [18, 19]. Macrophages exert a proinflammatory or an anti-inflammatory effect based on the microenvironment in different stages of ALI. Alveolar macrophages can shift to the predominant M1 phenotype in response to infection. M1 macrophages can act as the first line of defense against pathogenic microorganisms and lung injury, such as bacteria, viruses, and lung injury induced by ventilator [20], releasing various potent proinflammatory mediators, including IL-1β, TNF-α, IL-6, and IL-10 [21]. Therefore, M1 macrophages serve as a promoter in the early process of lung tissue damage in ALI. After pathogenic factors are eliminated, M1 phenotype may change into the anti-inflammatory M2 phenotype. M2 macrophages play an important role in lung tissue repair by limiting the levels of proinflammatory cytokines. M2 macrophages can also produce anti-inflammatory cytokines, such as IL-10 and IL-1 receptor antagonist, in response to Th2 cytokines [22]. Phagocytosis of apoptotic neutrophils by M2 macrophages further increases the levels of TGF-β1, which can eliminate inflammation [23]. In our study, we found large numbers of macrophages in ALI lung tissue.

CXCR4 is a member of the G protein-coupled receptor protein superfamily [24]. CXCR4 interacts with CXCL12 to initiate downstream signaling pathways. Then, CXCR4 plays a key role in modulating signal transduction, chemotaxis of inflammation cells, and maintaining the homeostasis of inflammatory responses [25]. Recent studies have found that in ALI mice, lung tissues develop bleeding and edema, and CXCR4 expression was significantly increased in various experimental models. CXCR4 could mediate neutrophil exudation and promote neovascularization [26–30]. Based on these data, the mechanism maybe CXCR4 is elevated after vascular endothelial cells and alveolar epithelial cells damaged. Then, CXCR4 could activate G protein-coupled signaling pathways and transcription factors, induce cell damage and apoptosis, and release a series of inflammatory factors. This process also damages lung tissue. In this study, we also found that the expression of CXCR4 was upregulated in macrophages after LPS stimulation, which suggested that CXCR4 may act as a target gene in the occurrence and development of ALI.

Furthermore, CXCL12/CXCR4 activation through multiple downstream pathways leads to increased cell proliferation and migration, such as NF-κB [31] and PI3K/Akt [32] pathway activation. CXCL12/CXCR4 can act on MAPK pathway through G protein and then act on its downstream ERK1/2 and FAK to cause cell chemotaxis and inflammatory cytokine accumulation [33–36].

In this study, we found that the expression of CXCR4 was significantly increased in macrophages of ALI mice models. This finding suggested that CXCR4 may regulate the function of macrophages. Therefore, we used siRNA interference technology to knockdown CXCR4. We found that the expression of IL-6 and TNF-α was obviously decreased, but IL-10 expression was increased compared to that of the control. Next, we investigated the mechanism by which CXCR4 knockdown decreases the expression of IL-6 and TNF-α in macrophages. The MAPK and NF-κB signaling pathways are the downstream signaling of LPS, so we detected the activation of these two pathways. We first detected the MAPK signaling pathway and discovered that the phosphorylation of p38 and Erk1/2 was significantly decreased. However, there was no obvious change in the phosphorylation of JNK. Then we also detected the NF-κB signaling pathway and found that the phosphorylation of p65 was also obviously increased. In conclusion, we proved that blocking CXCR4 may inhibit inflammatory cytokine production in macrophages by MAPK and NF-κB signaling pathway activation. This finding suggested that CXCR4 may participate in the development of ALI and aggravate pulmonary inflammation response. However, its specific effects and mechanisms need further research. Combined with previous studies, CXCR4 was shown to play a key role in occurrence and development of ALI induced by LPS, which may be a new target of ALI treatment.

## Conclusions

In conclusion, we have demonstrated that CXCR4 knockdown may suppress the function of macrophages by suppressing MAPK and NF-κB signaling pathway activation. This suggested that CXCR4 knockdown may have potential clinical value in treating ALI.

## Data Availability

Please contact the corresponding author for data on reasonable request.
